# Colonization of Grande Comore Island by a lineage of *Rhipicephalus appendiculatus *ticks

**DOI:** 10.1186/1756-3305-4-38

**Published:** 2011-03-17

**Authors:** Amina Yssouf, Erwan Lagadec, Annabelle Bakari, Coralie Foray, Frédéric Stachurski, Eric Cardinale, Olivier Plantard, Pablo Tortosa

**Affiliations:** 1 Centre de Recherche et de Veille sur les Maladies Emergentes dans l'Océan indien (CRVOI) -GIP CYROI- 2, rue Maxime Rivière, 97490 Ste Clotilde, France; 2 Ministère de l'agriculture, de la Pêche, de l'Environnement, de l'Energie, de l'Industrie et de l'Artisanat. Moroni. République des Comores; 3 FOFIFA- Département de Recherches Zootechniques et Vétérinaires (DRZV), Antananarivo, Madagascar; 4 Unité Mixte de Recherche Contrôle des Maladies Animales Exotiques Emergentes, CIRAD, Montpellier, France; 5 INRA, UMR 1300 BiOEpAR (Bio-agression, Epidémiologie et Analyse de Risques en santé animale), Nantes, F-44307 France; 6 LUNAM Université, Oniris, Nantes, F-44307, France; 7 Université de La Réunion. Fédération de recherche Environnement, Biodiversité et Santé. 15, avenue René Cassin, Ste Clotilde 97490 St Denis, France; 8 Centre National de la Recherche Scientifique, UMR5557 Ecologie Microbienne, Bât A. Forel, 43 bd du 11 novembre 1918, 69622 Villeurbanne CEDEX, France; 9 Plan National de Lutte contre le Paludisme, Moroni, République des Comores

## Abstract

**Background:**

Union of the Comoros suffered a severe East Coast Fever epidemic in 2004. *Rhipicephalus appendiculatus *was probably involved in pathogen transmission as this competent tick species, although previously absent from Comoros, was sampled on 4 animals on one geographical site during the epidemic. We carried out an entomological survey on all three islands of Union of the Comoros to establish cattle tick species distribution with a special emphasis on *R. appendiculatus*. We investigated *R. appendiculatus *intraspecific diversity as this species has been previously shown to be split off into two main cytoplasmic lineages with different ecology, physiology and vectorial competence. This survey also included sampling of live cattle imported from Tanzania to investigate the possibility of tick introduction through animal trade.

**Results:**

Our data show that Comoros cattle are infested with *Amblyomma variegatum*, *Rhipicephalus microplus *and *R. appendiculatus*. This latter species has established throughout Grande Comore but is absent from Anjouan and Moheli. Interestingly, 43 out of the 47 sequenced *R. appendiculatus *ticks belong to one single highly competent lineage while ticks from the other lineage where only found on imported cattle or on cattle parked at the vicinity of the harbor. At last, 2 ticks identified as *R. evertsi*, a species so far virtually absent on Comoros, were sampled on imported cattle.

**Conclusions:**

This survey shows that importation of live cattle is clearly a source of vector introduction in Comoros. The wide distribution of one highly competent *R. appendiculatus *lineage on Grande Comore, together with the absence of this species on the two neighbouring islands is in accordance with the rapid and disastrous spread of East Coast Fever epidemics on Grande Comore Island only. Whether the other *R. appendiculatus *lineage as well as *R. evertsi *species will succeed in establishing permanently on Grande Comore needs to be monitored.

## Introduction

The Comoros archipelago lies in the north entrance of the Mozambique Channel, roughly 180 miles East of Tanzania and 180 miles West of the North Western tip of Madagascar. The archipelago is composed of 4 islands, one of them, Mayotte, being under French administration. The islands of Grande Comore, Moheli and Anjouan, compose Union of the Comoros. In the last 10 years, the country has been affected by three successive epidemic waves whose causative agents were highly suspected to spread from East Africa. In 2002/2004, an East Coast Fever epidemic, a tick-borne disease transmitted by *Rhipicephalus appendiculatus*, led to the death of 10% of cattle on Grande Comore [1]. The isolated *Theileria parva *strains, once genotyped, were shown to be closely related to the strain Muguga, widely used for vaccination in East Africa. In the following year, a severe Chikungunya epidemic hit the country [2] before spreading to Reunion Island [3], then to most of the Indian Ocean islands and continental countries and eventually to Europe, where indigenous transmission was recorded in 2007 [4]. Finally, in 2008, a first case of human Rift Valley Fever was diagnosed in Mayotte Hospital Center in a child arriving from Union of the Comores [5]. This first human case was followed by several human deaths on Madagascar and partial sequence analysis showed that viral strains circulating in Madagascar were very similar to strains circulating in Kenya in 2006-2007 [6]. Only scarce epidemiological data are available as far as Comoros are concerned. However, phylogenetic studies carried out on Chikungunya [3], the timing of spreading of both Chikungunya and Rift Valley Fever viruses in the region together with *T. parva *genotyping in Grande Comore [1] strongly suggest that Comoros are a main entrance gate for pathogens introduced from East Africa. The emergence of these pathogens to the other island states probably depends on human and trade exchanges among these islands, as well as on the presence of competent vectors and putative reservoirs in the newly colonized areas. Therefore, entomological investigations on Comoros and the neighbouring islands, together with a dedicated surveillance of human and animal exchanges between East Africa and Comoros are of primary importance in controlling emerging vector-borne diseases in the whole South Western Indian Ocean region.

Comoros capital Moroni is located on Grande Comore. Most international trade goes through Moroni international airport or harbor and is then distributed to Anjouan and Moheli. The country has been importing goods and livestock from Tanzania since the year 2000 after the signature of a free trade bill between Union of the Comoros and Tanzania. It is interesting to note that most cattle movements inside Union of the Comoros unidirectionally target Grande Comore where traditional weddings require important bovine slaughtering. As a result, cattle imported from East Africa or reared in Grande Comore are only rarely sent to Anjouan and Moheli while cattle movements between Moheli and Anjouan and from both islands towards Grande Comore are common. These movements certainly have important implications in terms of vector and pathogens dissemination.

In this paper, we report a comprehensive entomological survey carried out on Union of the Comoros cattle. A special emphasis was put on *Rhipicephalus appendiculatus *species as a known vector of *Theileria parva*. Although only a few specimens were previously sampled on four animals at a single site [1], this tick species is suspected to have established permanent populations on Grande Comore since the East coast Fever epidemic was widespread in 2004. This species has not been previously described on either Moheli or Anjouan Islands. In this survey, ticks were collected from the whole country as well as on imported cattle arriving by boat from Tanzania into Moroni harbor. In addition to morphological identification of collected samples, we conducted molecular investigations to assess the intra-specific genetic diversity of the Comorian populations of *R. appendiculatus*. Indeed, former studies have shown that this species may be separated in two genetically distinct cytoplasmic lineages with marked different ecology and distribution [7-10]. Phylogenetic investigations were thus carried out on *R. appendiculatus *to identify their lineage and origin. The consequences of our findings on the epidemiology of tick-borne disease in the Comorian archipelago are discussed.

## Methods

### Study sites and tick sampling

All the islands of the archipelago are distributed 25 to 40 miles apart. Despite their volcanic origin, they differ markedly in their geographical and geological features: Grande Comore is the youngest island and its active Karthala volcano the highest summit (2361 m) of the archipelago. The porosity of its ground leads to the absence of watercourses while both Anjouan and Moheli exhibit developed hydrological systems. This geological diversity may have important consequences in terms of arthropod-borne diseases. Ticks were sampled throughout the three islands of Union of the Comoros during the rainy season, from January to February 2010. Sampling sites were randomly selected in the frame of a cattle surveillance program conducted by CRVOI (Centre de Recherche et de Veille sur les Maladies Emergentes dans l'Océan Indien). Fourteen sites were sampled on Grande Comore, 14 on Anjouan and six on Moheli. Altogether, 16 of the 17 districts of Comoros were sampled. On each farm, up to five animals, cows and goats, were sampled as follows: animals highly infested were first identified by a rapid visual check-up and examined; in the absence of highly infested animals, five were randomly chosen and carefully examined. All ticks were collected on animals with low infestation. Animals with more than 150 ticks were sampled as follows: up to 20 ticks were collected sequentially from feet, legs, anus, scrotum, udder, neck, ears and eyes. All ticks collected from one anatomical part on a single animal were pooled regardless of species and stadia. In the case of farms possessing less than five animals, an adjacent farm was investigated to complete the sampling.

Complementary to the sampling of ticks in farms, samples were collected from imported cattle parked in quarantine paddocks located in the vicinity of the harbor within 24 h after cattle landing, since it was not possible to sample ticks directly on board of merchant vessels. Cattle from three distinct vessels between 2009, December 12^th ^and 2010, February 11^th ^were studied. The name of the boat, the references of the trader were taken and each sampled cattle anonymously identified.

### Diagnosis and spatial analysis

Ticks were identified morphologically with the help of the key provided by Walker *et al*. [11]. Diagnosis was confirmed on some of the samples by using cytochrome oxydase 1 (*co1*) encoding a gene sequence widely used in barcoding projects [12]. The geographical positions of the collection sites were localized by a Global Positioning System. These coordinates were used to create maps with ArcGIS 9.3 management data software (ESRI, Union of the Comoros) by using sampling data (species and tick number) or *R. appendiculatus *haplotype number as inputs.

### Sequencing and phylogenetic analysis

DNA was extracted with Qiagen DNA and tissue kit following the instructions of the manufacturer except that an additional overnight proteinase K step was added to the provided protocol. Mitochondrial *co1 *locus was amplified by PCR with LCO1490 and HCO2198 primers as previously described [13]. Amplicons were directly sequenced after PCR purification and primers sequences were removed before alignments. In addition to the produced sequences, available *R. appendiculatus *sequences were downloaded from GenBank and added to the analysis (accession numbers: AF132833 from Zimbabwe, DQ859261-1, DQ859262-1, DQ859265-1, DQ859266-1, DQ901361-1, DQ901363-1 and DQ901364-1 from Zambia, DQ901356-1 from South Africa, DQ901357-1 from Grande Comore, DQ901358-1 and DQ901359-1 from Kenya, DQ901360-1, DQ901362-1 and DQ901363-1 from Rwanda). Two additional sequences, one for *R. turanicus *(DQ859260) and one for *R. evertsi *(AF132835) were included as outgroups. Alignments were conducted with ClustalW. However, no insertion-deletion was observed in the alignment file. Phylogenetic analyses were conducted in MEGA4, using the Neighbor-Joining method [14] and the Kimura 2-parameter method [15]. Bootstrap values were calculated with 10 000 replications. All positions containing missing data were eliminated only in pairwise sequence comparisons (Pairwise deletion option). Median spanning network was also constructed using version 4.5.1.6 of the Network software [16].

## Results

### Distribution of tick species in Union of the Comoros' cattle

**Figure 1:**
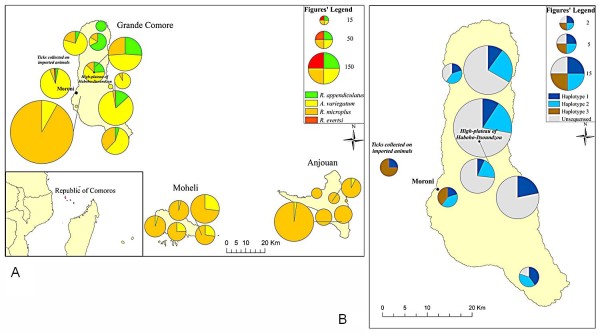
**Geographical distribution of cattle' ticks species and *R. appendiculatus *haplotypes on Union of the Comores**. (A) Geographic situation of Union of the Comoros and ticks species distribution. Circular diagrams indicate the proportion of each collected ticks species (*R. appendiculatus*, *A. variegatum*, *R. microplus *and *R. evertsi evertsi*); the diagrams' size is proportional to the total number of collected ticks. (B) Distribution of *R. appendiculatus *haplotypes 1, 2 and 3 in Grande Comore. Grey surfaces correspond to un-genotyped *R. appendiculatus *specimens and circular diagrams indicate the proportion of each haplotype.

The identification of 1986 collected ticks showed that cattle are infested with 3 tick species: *A. variegatum*, *R. appendiculatus *and *R. microplus*. Goats were sampled on Grande Comore and some of them were found to be infested with *A. variegatum *(data not shown). As shown in Table 1 and Figure 1-A, *R. microplus *is the most frequently occurring species on all three islands with a maximum in Anjouan where 98% of the collected ticks belong to this species. Our data show that *R. appendiculatus *species is widely distributed on Grande Comore but is absent on the neighboring Anjouan and Moheli islands, despite substantial sampling efforts to detect this species.

**Table 1:** Description of tick sample

	*A.variegatum N (%)*	*R.appendiculatus N (%)*	*R.microplus N (%)*	*R.evertsi*	Total
**Grande Comore**	480 (38,6)	122 (9,8)	642 (51,6)	0	1244
**Anjouan**	9 (2,3)	0	388 (97,7)	0	397
**Mohéli**	64 (18,6)	0	281 (81,4)	0	345
**Imported cattle**	120 (90.9)	4 (3)	6 (4.6)	2 (1.5)	132

This table shows the number and species distribution of ticks sampled throughout the 3 islands of Union of the Comoros as well as on the imported cattle.

### Tick species introduction through livestock trade

Each of the 3 investigated boats carried between 6 and 54 cows and altogether 132 tick specimens were collected. As shown on Table 1 and Figure 1-A, imported cattle display a different infestation pattern since they are mainly infested with *A. variegatum *although *R*. *appendiculatus *and *R. microplus *were also sampled. Two specimens belonging to *Rhipicephalus evertsi evertsi *species were sampled on imported cattle.

### Genetic characterization of the *Rhipicephalus *ticks found in Grande Comore

**Figure 2:**
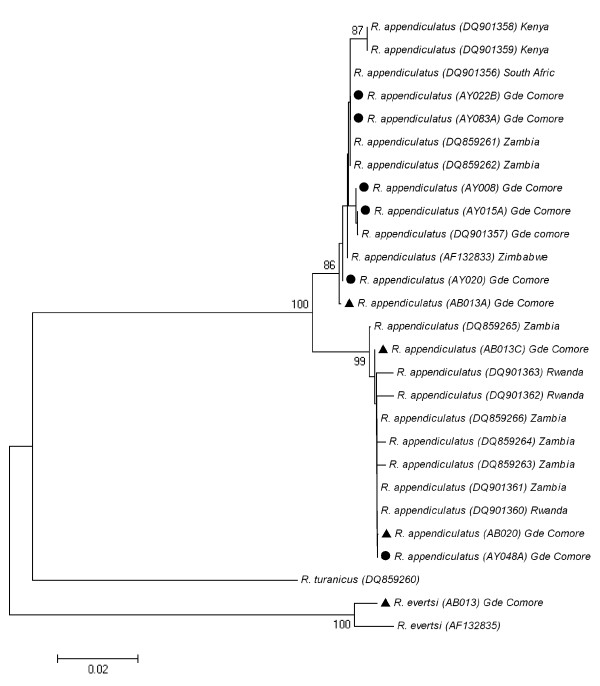
**Phylogenetic tree of *R. appendiculatus***. The evolutionary history was inferred using the Neighbor-Joining method [14]. New sequences provided by the present study are indicated by a black dot (ticks collected in farms) or a black triangle (ticks collected from imported cattle) in front of the sequence name (containing the accession number). The values above nodes correspond to bootstrap values, with only values superior to 80 indicated [20]. The tree is drawn to scale, with branch lengths in the same units as those of the evolutionary distances used to infer the phylogenetic tree. The evolutionary distances were computed using the Kimura 2-parameter method [15] and are in the units of the number of base substitutions per site. All positions containing missing data were eliminated only in pairwise sequence comparisons (Pairwise deletion option). There were a total of 793 positions in the final dataset. Phylogenetic analyses were conducted in MEGA4 [21].

**Figure 3:**
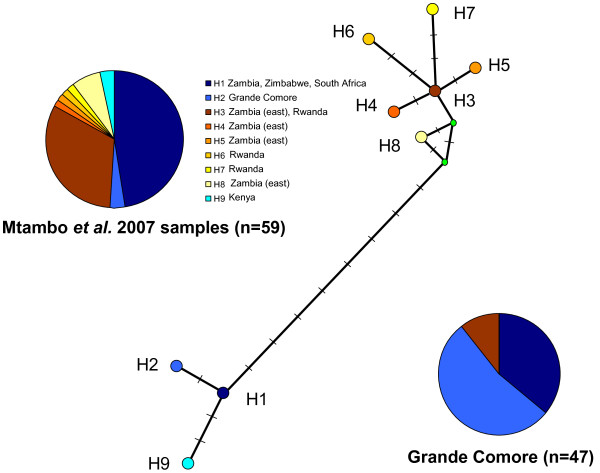
**Median network illustrating the relationships between the 9 mitochondrial haplotypes based on the *co1 *gene of *R. appendiculatus***. All those haplotypes were described in Mtambo et al. 2007 (except the haplotype 9, see text). Each circle corresponds to an haplotype, except the 2 smaller green circles that are « median Vectors » (corresponding to hypothesised sequences which are required to connect existing sequences within the network with maximum parsimony). Each orthogonal bar along the bold lines corresponding to a substitution in the data set. Pie-charts illustrate the frequency of the different haplotypes in the Mtambo et 2007 study [9], and in the present study, concerning only samples from Grande Comore.

Forty-three *R. appendiculatus *specimens collected throughout Grande Comore and six specimens sampled on imported cattle (4 *R. appendiculatus *and 2 *R. evertsi*) were sequenced for the *co1 *locus. The identification of the two ticks found in the quarantine paddocks as belonging to *R. evertsi *(AB002 and AB013) was confirmed by their *co1 *sequence. Among the *R. appendiculatus *sequences that were already available in GenBank, sequences range from 793 (AF132833 and AF132835) to 478 nucleotides sites. The new sequences obtained in the present study range from 633 to 699 nucleotides sites. The Neighbor-Joining phylogenetic tree based [8] on *co1 *sequences (Figure 2) shows the presence of two clades, an "eastern clade", supported by a 99% bootstrap value, gathering samples from eastern Zambia, Rwanda and Grande Comore, and a "southern clade" supported by a bootstrap value of 86% and gathering samples from southern Zambia, Zimbabwe, Kenya, South Africa and Grande Comore. When considering the alignment of 478 nucleotides sites described by Mtambo et al. 2007 [9], respectively six and three haplotypes can be recognized in the eastern and the southern clades. Within the southern clade, in addition to the previously described haplotypes H1 and H2, we have identified a new haplotype, that we have named haplotype H9 (see Figure 3) corresponding to the sequences DQ901358 and DQ901359 from Kenya. This new haplotype is due to the variable position number 261 and 339. The H1 and H2 haplotypes (with 36 and 53% of the individuals from Grande Comore respectively), are by far the most frequent haplotypes in Grande Comore. The H2 haplotype has not been found so far outside from this archipelago. When considering a longer alignment, we have been able to identify four new variable sites. On the basis of those additional variable sites, two new variants can be identified within the haplotype H1: H1a with an "A" at position 222 of AF132833 sequence from Zimbabwe - also found in AY020 specimen sampled in a farm on Grande Comore, and H1b with a "G" at this position, found in the 16 other H1 haplotype collected in Grande Comore. Among the six haplotypes found in the eastern clade, the H3 haplotype is the most common one and is represented by five ticks in our sampling (*i.e*. 11% of the individuals from Grand Comore): three from ticks collected on the imported cattle and two from ticks sampled in a farm located 1.5 mile away from the harbor.

## Discussion

Recent human or animal outbreaks have illustrated that the Comoros archipelago may have constituted a bridge for the colonization of pathogens in the Indian Ocean islands from East Africa. In this frame, entomological surveys dedicated at inventorying tick species may be especially useful to prevent future epidemic events. Investigating intra-specific genetic variability may also be used, especially if the genetic variants have different vectorial competence for a given pathogen agent, or can inform on the geographical origin of an introduced vector.

The present study has revealed that *A. variegatum*, *R. microplus *and *R. appendiculatus *are the three species infesting Comoros cattle. *R. microplus *is the most common species in the country while *R. appendiculatus *was found on Grande Comore Island only, where it is widespread. Such a distribution is in accordance with the spreading of East Coast Fever on the entire Grande Comore island during the 2002-2004 epidemic while no case was reported on Moheli and Anjouan. The actual distribution of *R. appendiculatus *on Comores relieves at least temporarily the threat for an East Coast Fever epidemic on Moheli, Anjouan as well as on the French administrated Mayotte Island.

Beside locally reared cattle, it appears that imported cattle are also infested with ticks, although displaying a different infestation pattern. These cattle are infested with *A. variegatum*, *R. microplus*, *R. appendiculatus *and *R. evertsi evertsi*. This latter species is the most widespread of all *Rhipicephalus *species in Africa [11] but was not previously described in Comoros. This species is a known vector for babesiosis as well as a potential vector for Crimean Congo Hemorrhagic Fever [17] and must therefore be considered as a potential threat. Although this species was not found outside from the cattle parked in the quarantine paddocks, whether or not this species will succeed in establishing on Comoros will need to be monitored.

Morphological, physiological and phylogenetical data support the existence of at least two distinct *R. appendiculatus *stocks representing two ecological groups [8-10,18], *i.e*. a group found in Southern and Eastern Africa (Southern stock, distributed in South Africa, Southern Zambia and Zimbabwe) and a second group distributed mainly in Eastern Africa (Eastern stock, found in parts of Burundi, Kenya, Tanzania and Uganda). Both stocks display major morphological, ecological and epidemiological differences [7]. Ticks from the southern stock are larger, display a unimodal phenology and obligatory diapause while ticks from the Eastern stock are smaller than their southern stock counterparts, display a bimodal phenology and require a photoperiod induction for diapause. Additionally, ticks from the southern stock were reported to be more competent for *T. parva *than ticks from Eastern stock [19]. Our phylogenetic tree, based on the mitochondrial gene cytochrome oxydase 1, clearly shows that 89% of the sequenced ticks from Grande Comore belong to the southern stock clade. Two ticks sampled on cattle within farms displayed an East African stock haplotype and were noteworthy collected just 1.5 mile off the Moroni harbor. Four *R. appendiculatus *specimens collected on imported cattle were shown to belong to both clades. Therefore, although both lineages are imported from East Africa, it seems that the eastern lineage has not established a stable and permanent population on Grande Comore. This difference in the colonization pattern of the two lineages may be due to a better fit of the ticks from the southern stock to the ecological conditions found on Grande Comore. Alternatively, this pattern might result from a founder effect. However, it is interesting to note that we observe a higher haplotypic and species diversity in imported cattle and in the harbor neighborhood. Such pattern rather supports a better adaptation of ticks from the southern stock to Grande Comore ecological conditions.

The geographical origin of the H2 haplotype, only found in Grande Comore to date, may be identified in coastal areas of East Africa by extensive sampling surveys. Moreover, additional and more discriminatory genetic markers could be developed to trace the route of colonization followed by *R. appendiculatus *in the Comoros archipelago.

## Competing interests

The authors declare that they have no competing interests.

## Authors' contributions

AY sampled ticks throughout Comoros, identified morphologically each specimen with the help of EL who additionally performed most of the sequencing work. AB sampled ticks on imported cattle. CF prepared the spatial materials depicted in Figure 1. FS elaborated sampling protocol and confirmed tick identification whenever needed. EC participated in protocol design; OP performed the phylogenetic analysis and contributed substantially to the manuscript draft. PT supervised the work and wrote the manuscript. All authors read and approved the final manuscript.

## Aknowledgements

We wish to thank Ahmed Ouledi and the University of Comoros for providing substantial logistical help and for fruitful discussions. We are thankful to Sébastien Girard and Fédération Nationale des Eleveurs Comoriens (FNAC) for coordinating sampling throughout the country. Amina Yssouf grant was provided by French Government through Préfecture de La Réunion. This work was financed by FEDER Réunion, Programme Opérationnel de Coopération Territoriale (2007-2013), "Faune Sauvage-Océan Indien #31189" and "Animal Risk #290038".
